# Dispersal Modifies the Diversity and Composition of Active Bacterial Communities in Response to a Salinity Disturbance

**DOI:** 10.3389/fmicb.2018.02188

**Published:** 2018-09-19

**Authors:** Dandan Shen, Silke Langenheder, Klaus Jürgens

**Affiliations:** ^1^Section of Biological Oceanography, Leibniz Institute for Baltic Sea Research, Warnemünde, Germany; ^2^Department of Ecology and Genetic/Limnology, Evolutionary Biology Centre, Uppsala University, Uppsala, Sweden

**Keywords:** bacteria, dispersal, environmental heterogeneity, transplant experiment, community similarity

## Abstract

Dispersal can influence the response of bacterial communities to environmental changes and disturbances. However, the extent to which dispersal contributes to the community response in dependence of the character and strength of the disturbance remains unclear. Here, we conducted a transplant experiment using dialysis bags in which bacterioplankton originating from brackish and marine regions of the Saint Lawrence Estuary were reciprocally incubated in the two environments for 5 days. Dispersal treatments were set-up by subjecting half of the microcosms in each environment to an exchange of cells between the marine and brackish assemblages at a daily exchange rate of 6% (v/v), and the other half of microcosms were kept as the non-dispersal treatments. Bacterial 16S rRNA sequencing was then used to examine the diversity and composition of the active communities. Alpha diversity of the marine communities that were exposed to the brackish environment was elevated greatly by dispersal, but declined in the absence of dispersal. This indicates that dispersal compensated the loss of diversity in the marine communities after a disturbance by introducing bacterial taxa that were able to thrive and coexist with the remaining community members under brackish conditions. On the contrary, alpha diversity of the brackish communities was not affected by dispersal in either environment. Furthermore, dispersal led to an increase in similarity between marine and brackish communities in both of the environments, with a greater similarity when the communities were incubated in the brackish environment. These results suggest that the higher initial diversity in the brackish than in the marine starting community made the resident community less susceptible to dispersing bacteria. Altogether, this study shows that dispersal modifies the diversity and composition of the active communities in response to a salinity disturbance, and enables the local adjustment of specific bacteria under brackish environmental conditions.

## Introduction

Dispersal acts as a link between local and regional community dynamics within the framework of metacommunities (Mouquet and Loreau, 2003; Leibold et al., 2004). Both theoretical predictions (Loreau and Mouquet, 1999; Mouquet and Loreau, 2003) and experimental studies (Matthiessen and Hillebrand, 2006; Lindström and Östman, 2011; Declerck et al., 2013) have focused on how dispersal alters community diversity and composition. For microorganisms, the degree to which dispersal contributes to changes in community properties depends on (i) the magnitude of the dispersal rates (Lindström and Östman, 2011; Declerck et al., 2013; Souffreau et al., 2014; Berga et al., 2015), (ii) the initial diversity of the communities undergoing dispersal (Zha et al., 2016), and (iii) the source of the immigrants (Comte et al., 2017). In natural systems, and particularly in aquatic systems, the passive migration of cells often occurs in the setting of a change in environmental conditions, (Lindström and Langenheder, 2012; Rillig et al., 2015). This complicates the unraveling of the effect of dispersal from that of contemporary environmental conditions on the assembly of emergent bacterial communities. Only a few experimental studies have assessed the direct effects of dispersal, via the exchange of microorganisms, on the activity and overall structure of bacterial communities (Lindström and Östman, 2011; Severin et al., 2013). However, they did not consider how different environments affect the importance of dispersal on bacterial community composition and whether dispersal varies among bacterial taxa.

Dispersal can drive species coexistence via competition–colonization tradeoffs (Lowe and McPeek, 2014). Species interactions play a large role in regulating the colonization ability of immigrants and lead to the coexistence of local communities (Chesson, 2000). Bacterial taxa differ in their ability to colonize new environments and in their ability to compete with other community members (Livingston et al., 2012). Nonetheless, species interactions are important for most microorganisms that are likely to disperse passively (i.e., wind-blow or water flows) (Martiny et al., 2006; Nemergut et al., 2013), given that dispersal is random with respect to taxon identity. The variability at both the community and the population level after dispersal is therefore assumed to reflect the cumulative effects of competition and colonization on species coexistence.

Dispersal also influences the response of bacterial communities to disturbances or environmental perturbations (Shade et al., 2012). Without immigration, bacterial communities respond to disturbances mainly through short-term physiological acclimations (Martiny, 2015). This may lead to a decrease in total cell abundances (Baho et al., 2012) or to the proliferation of some bacteria from the “seed banks” (Jones and Lennon, 2010). However, in the presence of dispersal, immigrating bacteria can colonize niches opened up by disturbances (Baho et al., 2012; Vuono et al., 2016; Comte et al., 2017), and fulfill the essential functions that were performed by taxa being lost due to the disturbances (Székely and Langenheder, 2017). Yet, the extent to which dispersal contributes to the community response following a disturbance varies depending on the character and strength of the disturbance remains unclear.

Salinity is an important determinant of bacterial community composition across global scales (Lozupone and Knight, 2007). In aquatic systems, numerous studies have cataloged bacterial taxa-specific changes in abundances along a salinity gradient (e.g., Bouvier and del Giorgio, 2002; Herlemann et al., 2011). A differential distribution of bacterial taxa along a salinity gradient or in estuaries implies that some taxa are vulnerable to altered salinity. The ongoing salinization of coastal habitats or basins due to climate changes and anthropogenic activities underlines the need to better understand the mechanisms by which salinity influences bacterial communities (Herbert et al., 2015; Mohrholz et al., 2015). However, little is known about the fate of immigrant taxa transported from different salinity conditions and the impact of competition with local communities. The fate of dispersed communities may depend on the niche breath of the immigrants, because changes in salinity may favor habitat generalists with ecological versatility and a broad salinity tolerance (Székely et al., 2013). Thus, characterizing the diversity and composition of dispersed communities following a salinity change will enhance our ability to predict the ecological consequences for a given microbial community under saltwater intrusion scenarios.

The main aim of our study was to examine how the responses of estuarine bacterioplankton communities to minor salinity changes are modified by dispersal. To address this, we implemented a full-factorial experiment using dialysis bags in which brackish and marine bacterioplankton originating from two sites within the St. Lawrence Estuary (SLE) were incubated under the respective environmental conditions from both sites, with and without dispersal of cells between the two inoculum sources. We used Illumina sequencing of 16S rRNA to specifically target the response of the active bacterial communities before and after dispersal events, because RNA provides a better indicator for extant microbial viability than DNA (Keer and Birch, 2003) and thus excludes possible confounding effects of intact but inactive cells (Nielsen et al., 2007). We hypothesize that (i) dispersal increases community diversity in the communities exposed to a salinity change; (ii) dispersal influences the compositional response of the communities to a change in salinity by introducing taxa able to thrive under the new environmental conditions; (iii) the importance of dispersal events differs among bacterial phylogenetic groups.

## Materials and Methods

### Study Site and Experimental Setup

The SLE was a suitable aquatic system for our study, as its microorganisms are not only exposed to fluctuations in salinity but are also transported via currents or tidal events (Mucci et al., 2011; Dinauer and Mucci, 2017). Surface water (depth: 3 m) from two regions of SLE was collected using a Rosette sampler: the Gulf of St. Lawrence (47° 11.1547′N, 59° 32.2932′W; salinity ∼30.35 psu), on August 26, 2015, and the Lower SLE (48° 38.3388′N, 68° 37.9090′W; salinity ∼24.29 psu), on August 30, 2015. For simplicity, the two sampling locations are referred to in the following as ‘marine’ and ‘brackish’ sites, respectively. Water from these sites served as the source for both the medium and the inoculum. Approximately 200 L of the sampled water from each site was then filtered through a 200 μm mesh into 30 L carboys to remove large zooplankton. The medium was subsequently filtered through 142 mm GF/F filters (Whatman, Dassel, Germany) to remove protists. The microbial inoculum was prepared by further filtering the <200 μm water through a 25 μm mesh to remove large phytoplankton. The inoculum and medium originating from the marine site were stored in a constant temperature room at 4°C in the dark for 4 days until initiation of the experiment. The inoculum and medium from the brackish site were collected at the day when the experiment was started (August 30) and stored in the constant temperature room where the experiment was later performed (see below).

**FIGURE 1:**
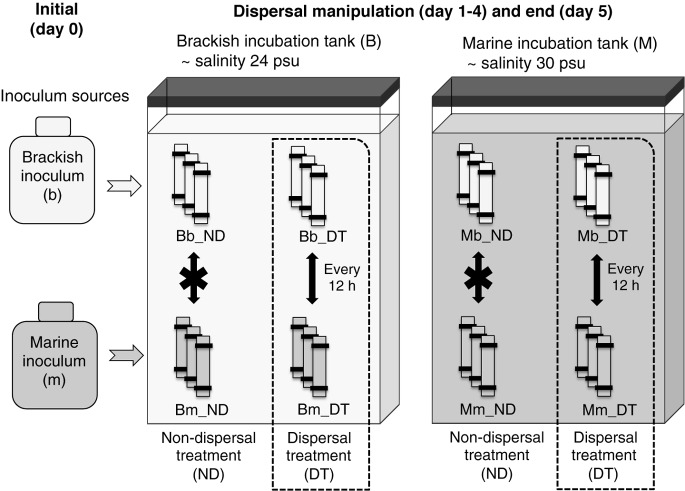
Experimental setup. Triplicate microcosms consisting of dialysis bags containing either a brackish or marine inoculum were reciprocally incubated in brackish and marine environments. B and M represent the brackish and marine incubation environments used in the incubations, respectively, and b and m represent the source (origin) of the initial brackish and marine inocula, respectively. ND indicates the treatments without dispersal, and DT indicates the treatments with dispersal. Combinations of these letters describe the particular incubation environment for the microcosms, the inoculum source, and the presence or absence of dispersal. For example, Bm_DT indicates microcosms in which the marine inoculum (m) was incubated in the brackish environment (B) and subjected to dispersal (DT).

Dialysis tubing with a molecular weight cutoff of 12–14 kDa (Spectrum Laboratories, Rancho Dominguez, CA, United States) was used to inoculate the microbial communities, as it ensured the free exchange of dissolved organic matter and nutrients without allowing the movement of microorganisms (protists, bacteria, viruses). Our choice of dialysis bags allowed the majority of organic molecules to diffuse efficiently, as the molecular mass of DOM in aquatic environments is typically smaller than 5 kDa (Wu et al., 2003; Nebbioso and Piccolo, 2013). Dialysis tubing pieces (45 cm long) were rinsed thoroughly 24 h before use, soaked in Milli-Q water overnight, and rinsed again. All inocula and media were acclimated to a constant temperature of 19°C for 12 h before setup of the experiment on August 30. Each dialysis bag was filled with 1.5 L of either brackish or marine inoculum and two incubation tanks were filled with ∼95 L of the brackish or marine medium, respectively. The dialysis bags were then placed in the tanks. The incubations were carried out in triplicates (total of 30 microcosms) for 5 days (**Figure 1**). The short duration was used in order to minimize the formation of biofilm on the surfaces of the bags, so that the permeability of the dialysis bags was maintained as much as possible during the course of the experiment. Salinities were measured and found to have equalized between the dialysis bags and incubation tank in <12 h. The experiment was implemented at constant temperature (19°C), in the dark and with no aeration.

The dispersal manipulation was initiated immediately after the salinity had equalized (12 h after the start of the incubation), by exchanging 50 mL of water by pipetting between half of the dialysis bags inoculated with the brackish and marine inocula twice per day (every 12 h), representing a daily exchange of 6% of each microcosm volume (dispersal treatment, DT, **Figure 1**). Our intention was to simulate a scenario that may occur in an estuary where marine and brackish communities are frequently mixed via tides or aerosols. The exchange of microorganisms was carried out between pairwise replicates. For example, in the brackish incubation tank, rep 1 of ‘Bb_DT’ exchanged with rep 1 of ‘Bm_DT (**Figure 1**), with the same procedure used for each pair of rep 2 or rep 3, correspondingly. Here and in the following, the capital letter B or M refers to the brackish or marine incubation environment, and the lower case letter b or m to the brackish or marine inoculum. For the treatments without dispersal, there was no exchange of microorganisms between pairwise replicates. However, to maintain the same level of physical disturbance in all microcosms, the remaining microcosms were subjected to the same action: pipetting water out from and into the same dialysis bag, but without exchanging microorganisms among the bags (non-dispersal treatment, ND, **Figure 1**). All dialysis-bag microcosms were destructively sampled on day 5 of the incubation (that is, 12 h after the last dispersal manipulation on day 4). One replicate of treatment Bb_ND was not included in all analyses due to water loss during sampling.

### Sampling and Sample Analysis

#### Microbial Cell Abundances

Samples were taken from the initial microbial inocula (day 0) and all microcosms (day 5) for determinations of microbial cell abundances. For bacterial abundance, 4 mL were preserved with formaldehyde at a final concentration of 2%, and immediately flash frozen in liquid nitrogen until used in the flow cytometry measurement. Cells in the samples were stained using 10x SYBR Green I (Life Technologies, Darmstadt, Germany) and then counted in a FASCalibur flow cytometer (Becton Dickinson, Fremont, CA, United States), as described elsewhere (Gasol and del Giorgio, 2000).

In addition, the cell abundance of protists was determined using epifluorescence microscopy, according to Weber et al. (2012) with minor modifications. Briefly, 10 mL of sample was fixed with formaldehyde at a final concentration of 2% and stored at 4°C. After ∼4 h, the fixed samples were filtered onto 0.8-μm, 25-mm black filters (Whatman, Dassel, Germany), which were then stored at −20°C until further processing. For cell enumeration, cells on the filters were stained with 4’,6-diamidin-2-phenylindol and three randomly selected fields of view were inspected at 63× magnification using a Zeiss Axioskop 2 mot plus microscope (Zeiss, Oberkochen, Germany). Technical triplicates were established for each sample.

#### Water Physiochemical Analyses

Conductivity was measured together with inorganic nutrients and dissolved organic carbon (DOC) concentrations. The samples were taken from the initial inocula and all microcosms at the end of the experiment. Briefly, 15-mL samples were filtered through GF/F filters (Whatman, Dassel, Germany) for measurements of NO_3_^−^, NO_2_^−^, PO_4_^3-^, NH_4_^+^, and SiO_2_^−^ concentrations using a colorimetric method according to Grasshoff et al. (1999) and a Seal Analytical QuAAtro automated nutrient analyzer (SEAL Analytical GmbH, Norderstedt, Germany). DOC was measured by filtering 20 mL of the samples through combusted GF/F filters (Whatman, Dassel, Germany), which were then analyzed on a TOC-VCPH TOC Analysator (Shimadzu Europe GmbH, Duisburg, Germany). **Supplementary Table S1** summarizes the main chemical and biological measurements of the water samples.

#### Active Bacterial Community Composition

Active bacterial communities were analyzed from two of the initial microbial inocula and all microcosms on day 5. Water samples (1.5 L) from the initial inocula and from each dialysis bag (∼1.4 L) were filtered through 0.22 μm pore size filters (Millipore, Darmstadt, Germany), which were immediately flash-frozen in liquid nitrogen and stored at −80°C until nucleic acid extraction. The RNA from 29 samples was extracted using the RNeasy mini kit (Qiagen, Hilden, Germany) according to the manufacturer’s protocol. Genomic DNA in the RNA extracts was removed by DNase treatment using the TURBO DNA-free kit^TM^ (Invitrogen, Darmstadt, Germany). DNase-treated RNA was tested for traces of DNA by PCR amplification using the bacterial 16S rRNA gene primers of two sets: comf1/r2ph (Stolle et al., 2011) and 341f/805r (Herlemann et al., 2011). The PCR products were visualized on 1.2% agarose gels. The above-described DNase treatment and the tested PCR steps were repeated until no positive DNA amplification was detected. The RNA extracts were further purified using the RNA Clean and Concentrator^TM^-5 kit (Zymo Research, Irvine, CA, United States), and the quality and concentration then checked on an Agilent 2100 Bioanalyzers. The purified RNA was reverse transcribed using the iScript cDNA synthesis kit (Bio-Rad, Munich, Germany). The resultant cDNA from all samples was PCR amplified using the primers 341f/805r (Herlemann et al., 2011), which allowed determination of the metabolically active fraction of the bacterial communities (Blazewicz et al., 2013) that responded to the experimental conditions and dispersal treatments. The amplicons were sequenced using the Illumina Miseq system (2 × 300 base pairs) at the LGC sequencing center (Berlin, Germany).

### Sequence Processing

Sequences were processed using MOTHUR v.1.36.1 according to the Miseq SOP, with minor customized modifications^1^ (assessed: May 1st, 2016) (Kozich et al., 2013). Paired-end sequences were merged. The sequences were then quality filtered such that any reads with length <400 nt, ambiguous bases >0, and a homopolymer length >8 were removed from further analysis. The remaining sequences were aligned with those in the SILVA v123 reference database; sequences that did not align to the correct region were eliminated. Noise in the sequences was further reduced using pre-clustering; the resulting sequences were screened for chimeras using UCHIME (Edgar et al., 2011). A Bayesian classifier was used to classify the sequences against the Ribosomal Database Project (Wang et al., 2007). Only classifications with a bootstrap cutoff value >80% were included in the analyses. All Archaea, Eukaryota, chloroplasts, mitochondria, and unknown sequences were removed from the sequence dataset. Finally, the sequences were clustered according to their taxonomy and assigned to operational taxonomic units (OTUs) at a 3% dissimilarity level using the average neighbor method. Singletons (OTUs with only one sequence across all libraries) were also discarded. For downstream analyses, the sequences were subsampled to 11,074 sequences (the size of the smallest library; see **Supplementary Table S1** for details) across 29 samples using the R script described in Zha et al. (2016). The FASTQ files and associated metadata have been deposited and are publically available at the European Nucleotide Archive^2^ under the accession number PRJEB23259.

### Statistics

Three-way ANOVAs were used to test the effects of dispersal, incubation environment, inoculum source, and their interactions on microbial cell abundances, Shannon diversity, species richness, and the evenness of the bacterial communities at the end of the experiment (day 5). All three factors served as fixed effects in all linear models that were tested by ANOVA. To assure the fulfillments of the assumptions of the ANOVA and when data needed to be transformed, the normal distribution of the residuals of the linear models was tested using the Shapiro–Wilk normality test. The homogeneity of variance was tested using Levene’s test using the package ‘car’ (v2.1-4). Data were log transformed if necessary to fulfill ANOVA requirements. In case of significant effects, Welch’s *t*-test, used under conditions of unequal variances, was carried out to further explore the differences between ND and DT for the brackish and marine communities, as well as the differences between the two initial microbial inocula. Realized species richness (S.Obs) and Shannon diversity (H) were computed from the average of each of 100 iterations using the ‘vegan’ R packages (v2.4-1; Oksanen et al., 2016). Realized evenness was calculated from the quotient of H/ln S.Obs.

Differences in bacterial community composition were visualized using non-metric multidimensional scaling (NMDS) based on the Bray–Curtis dissimilarity metric and by fitting environmental variables (i.e., salinity, nutrient content and protist abundance) to the ordination. Significant differences in the between-community variation among ND and DT of each environment were analyzed based on the test of homogeneity of multivariate dispersions (beta-dispersion) (Anderson, 2006). The effects of dispersal, incubation environment, inoculum source and their interactions on bacterial community compositions in all microcosms were analyzed using three-way permutational multivariate analyses of variance (PERMANOVA) (Anderson, 2001). A large fraction (52.23%) of the variance in the differences in communities could be attributed to the inoculum source. This variance was therefore excluded to improve the estimates of dispersal and incubation environment, by performing two-way PERMANOVA tests separately for the brackish and marine communities.

Three-way ANOVAs were also used to test the effects of dispersal, incubation environment, inoculum source, and their interactions on the abundances of bacterial phyla/classes or order/families. Normality and homogeneity of variance were checked as described above, arcsine square-root-transforming the data if necessary to fulfill the requirements of the ANOVA.

The occurrence patterns of abundant OTUs (mean relative abundance > 1% in any microcosm) were explored using a hierarchical analysis with Pearson’s correlation to cluster those OTUs with similar relative abundances. The dendrograms grouped taxa according to their occurrence patterns, without any information on phylogeny. Heatmaps with color gradients were used to present the trend in the relative abundance of each OTU. Among the abundant OTUs, those with a potentially high dispersal ability (termed ‘abundant dispersers’) in each environment were determined as well by identifying the OTUs that were present in the pool of abundant OTUs in DT, but were absent from either brackish or marine communities in ND. Although defining OTUs with good dispersal capabilities based only on the abundant OTU pool is somewhat arbitrary, it does offer more information than provided by taxonomy or functional capacity (Lindström and Langenheder, 2012). All statistical analyses and data visualization were performed in R (v3.4-0).

## Results

### Microbial Cell Abundance

**FIGURE 2:**
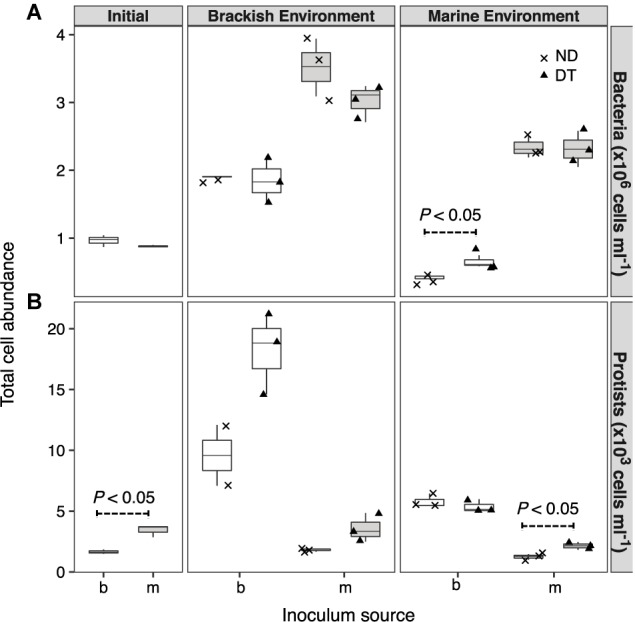
Cell abundances of bacteria **(A)** and protists **(B)** at the beginning of the experiment and after incubation in a brackish or marine environment for 5 days. The source of the initial microbial inoculum (*x*-axis) is indicated as brackish (b, white) or marine (m, gray). ND, non-dispersal treatment (×); DT, dispersal treatment (triangles). Significant differences at *P* < 0.1 determined in the corresponding Welch’s *t*-tests are shown in the figure.

Both the inoculum source and the environment, but not dispersal, had significant effects on bacterial abundances (three-way ANOVA, source: *F* = 224.57, *P* < 0.001; environment: *F* = 128.78, *P* < 0.001; **Supplementary Table S2**). However, the interaction between dispersal and environment marginally affected bacterial abundance (three-way ANOVA, *F* = 3.14, *P* < 0.1). This effect was most pronounced for the transplanted brackish communities under marine conditions, with higher cell abundances in DT (**Figure 2A**). The abundances of protists were significantly higher in the initial marine than in the initial brackish inoculum (**Figure 2B**). Dispersal, environment, and the inoculum source individually influenced protist abundance at the end of the experiment (**Supplementary Table S2**). Protist abundance was also significantly affected by the interaction between dispersal and environment (three-way ANOVA, *F* = 6.85, *P* < 0.05; **Supplementary Table S2**). In addition, a higher abundance was observed in DT for the marine communities grown in their native environment (i.e., incubation environment and inoculum source were matched with regard to water origin) (**Figure 2B**).

### Alpha Diversity

**FIGURE 3:**
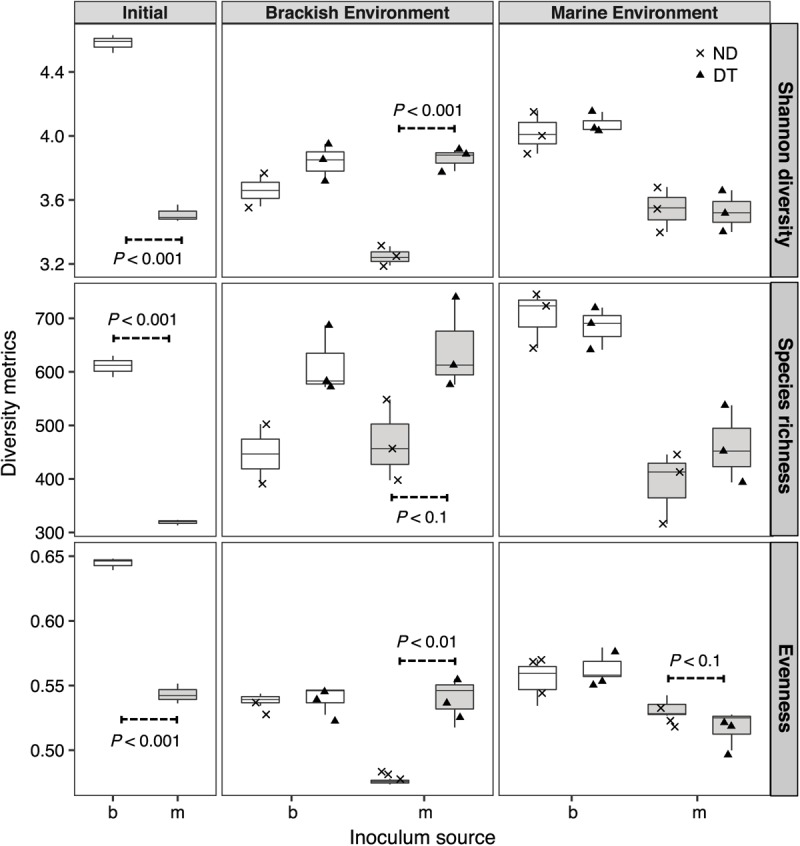
Shannon diversity, realized richness, and evenness of the active bacterial community at the beginning and end of experiment (day 5). The source of the initial microbial inoculum (*x*-axis) is indicated as brackish (b, white) or marine (m, gray). ND, non-dispersal treatment (×); DT, dispersal treatment (triangles). Significant differences at *P* < 0.1 determined in the corresponding Welch’s *t*-tests are shown in the figure.

Shannon diversity was significantly higher in the initial brackish than in the initial marine inoculum (**Figure 3**). The results of the three-way ANOVAs showed that dispersal, environment, and inoculum source influenced Shannon diversity, and both realized species richness and evenness (**Supplementary Table S2**). However, the interaction of these three factors resulted in weaker effects on richness and evenness (**Supplementary Table S2**). Dispersal resulted in greater Shannon diversity, richness and evenness in the brackish environment for the marine communities (**Figure 3**). In the case of brackish communities, the measured diversity metrics of ND and DT did not significantly differ in either environment (**Figure 3**).

### Beta Diversity

**FIGURE 4:**
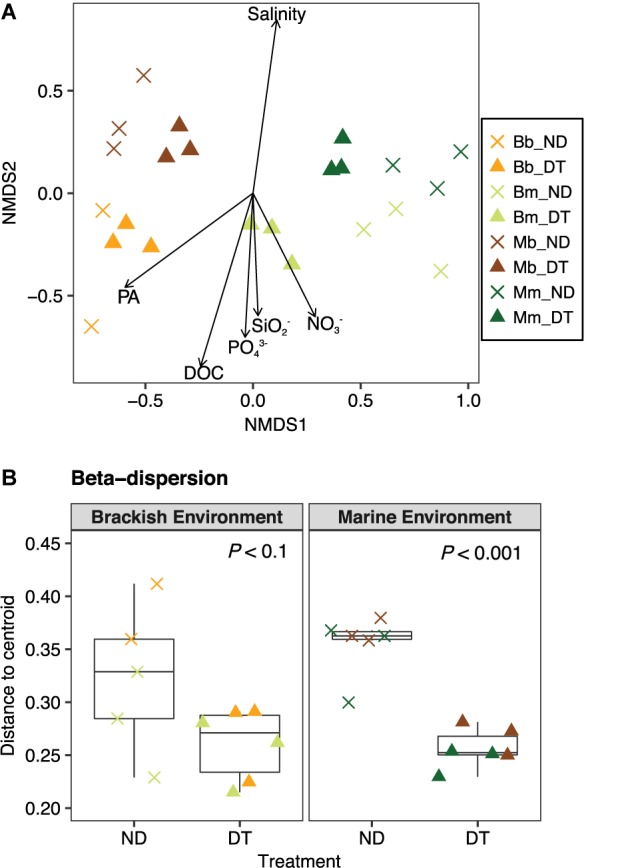
Beta diversity of the active bacterial communities. **(A)** Differences in the bacterial communities at the end of the experiment as determined by NMDS ordination. The particular combination of incubation environment and inoculum source is color-coded: b inoculum in the B environment is (Bb: orange), m inoculum in the B environment (Bm: olive green), b inoculum in the M environment (Mb: brown), and m inoculum in the M environment (Mm: dark green). ND, non-dispersal treatment (×); DT, dispersal treatment (triangles). The strength of the statistically significant (*P* < 0.05) explanatory environmental variables is shown with solid arrows (for explanatory values of the environmental variables to differences in the communities, see **Supplementary Table S2**). DOC, dissolved organic carbon; PA, protist abundance. **(B)** Beta-dispersion illustrating the mean differences in the variation (i.e., distance to centroid) between communities in ND and DT for the brackish and marine environments. Significant differences at *P* < 0.1 obtained from the corresponding Welch’s *t*-tests are shown in the figure.

The active bacterial communities became more similar in DT than in ND after 5 days of incubation, even though communities were mainly clustered according to the inoculum source (of marine or brackish origin, **Figure 4A**). The results of the PERMANOVA test showed that the variation in the beta diversity among all microcosms was significantly explained by the inoculum source (52.23%), followed by environment (9.32%), dispersal (3.50%), and the interactions between any of the two factors (2.92–6.62%) (**Supplementary Table S3A**). In the microcosms with a brackish inoculum and in those with a marine inoculum, the interaction between dispersal and environment was significant only for the marine but not the brackish communities and explained 14.35% of the total variance (two-way PERMANOVA, *P* < 0.05; **Supplementary Table S3B**).

To explore the relationship between the variability in the active community composition and incubation environment, the variables salinity, nutrient and protist concentrations were plotted in the NMDS ordination as fitting environmental variables. The variation in community composition among all microcosms correlated with differences in salinity, which were higher in the marine environment, but were also apparently related to other environmental factors, such as protist abundance, DOC, DOC, PO_4_^3−^, SO_2_^−^, and NO_3_^−^, which were higher in the brackish environment (**Figure 4A** and **Supplementary Tables S1**, **S4**).

To better understand the between-community variation in dependence of dispersal for each incubation environment, we performed beta-dispersion analysis to determine the mean differences in community dissimilarity among ND and DT. Dispersal generally led to a decrease in between-community variation, which was more apparent in the marine than in the brackish environment (**Figure 4B**).

### Taxon-Specific Responses to Dispersal

**FIGURE 5:**
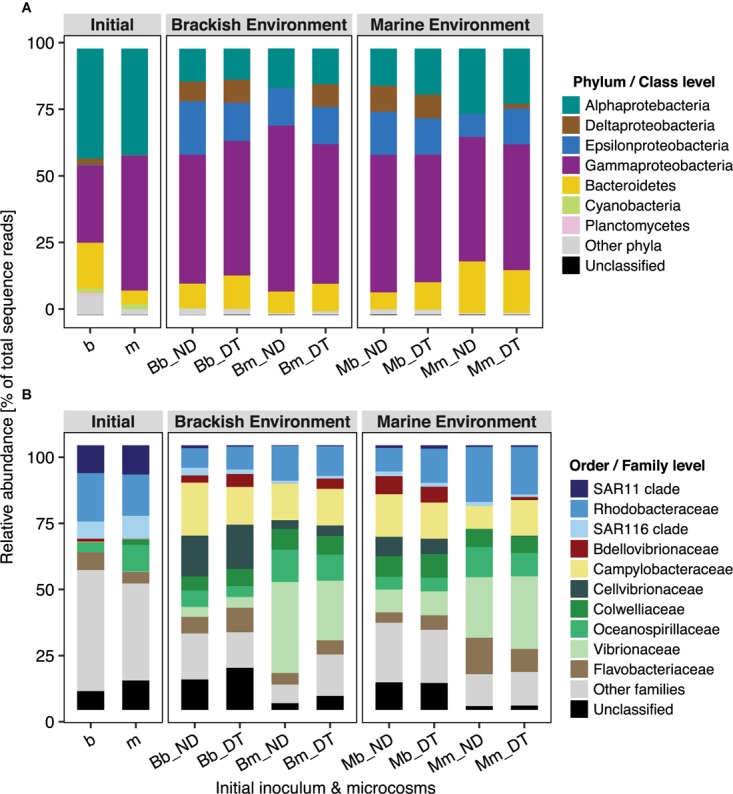
Taxonomic composition of bacterial communities in microcosms at the phylum/class **(A)** and order/family **(B)** levels. Relative abundance was calculated from the normalized reads, i.e., the percentage of total sequence reads, and is presented as the average value of triplicate samples (expect duplicate samples from Bb_ND microcosms). ‘Unclassified’ indicates OTUs that could not be assigned taxonomically at the phylum/class or order/family level.

Active bacterial communities were dominated by taxa assigned to Alpha-, Gamma-, and Deltaproteobacteria, and to Bacteroidetes (**Figure 5A**), together contributing ≥80% of total sequence reads. Three-way ANOVA tests showed significant differences in the abundances of several bacterial phyla or classes (**Supplementary Table S5**). Dispersal led to a significant increase in the relative abundance of Deltaproteobacteria but had weaker effects on the relative abundance of Alphaproteobacteria, Epsilonproteobacteria, and Bacteroidetes, depending on the inoculum source (**Figure 5A** and **Supplementary Tables S5A–C,E**), and no effects on the relative abundance of Gammaproteobacteria (**Supplementary Table S5D**).

Not all of the families/clades affiliated with particular phyla/classes responded identically to dispersal, except for *Bdellovibrionaceae* (Deltaproteobacteria) and *Campylobacteraceae* (Epsilonproteobacteria) (**Figure 5**). Overall, the interactive effects of dispersal and environment were more apparent for family-level than for the phylum/class-level responses (**Supplementary Table S5**). The interactions showed a stronger effect of dispersal for some families in the brackish environment. For example, dispersal resulted in a decrease in the relative abundances of *Oceanospirillaceae* (although not statistically significant) and *Vibrionaceae* in the brackish environment, independent of the inoculum source (**Figure 5B** and **Supplementary Table S5D**). Interestingly, *Bdellovibrionaceae* in the marine communities were present at low (relative) abundances in the brackish environment, but in the absence of dispersal, not at all in the native environment (**Figure 5B**); they were, however, significantly enriched in DT. The abundance of the SAR11 clade was marginally influenced by the interactions between dispersal and environment (**Supplementary Table S5A**), but without a clear pattern. By contrast, the interaction did not significantly influence the SAR116 clade (**Supplementary Table S5A**), *Cellvibrionaceae*, or *Colwelliaceae* (**Supplementary Table S5D**). Neither dispersal nor incubation environment had effects on the abundances of *Flavobacteriaceae*, whereas their interactive effect was significant (**Supplementary Table S5E**). This suggested that, at least in one of the environments, both the brackish and the marine communities had a unique response to dispersal. For example, the abundances of members of *Flavobacteriaceae* showed a slight but significant increase in response to dispersal in the brackish incubation environment (**Figure 5B**). In the case of *Rhodobacteraceae* and *Campylobacteraceae*, the interaction between dispersal and inoculum source significantly influenced their relative abundances: the former one decreased when it originated from the marine and the latter one when it originated from the brackish inoculum (**Figure 5B** and **Supplementary Tables S5A,C**, respectively).

### Occurrence Patterns of Abundant OTUs and the Identification of ‘Abundant Dispersers’

**FIGURE 6:**
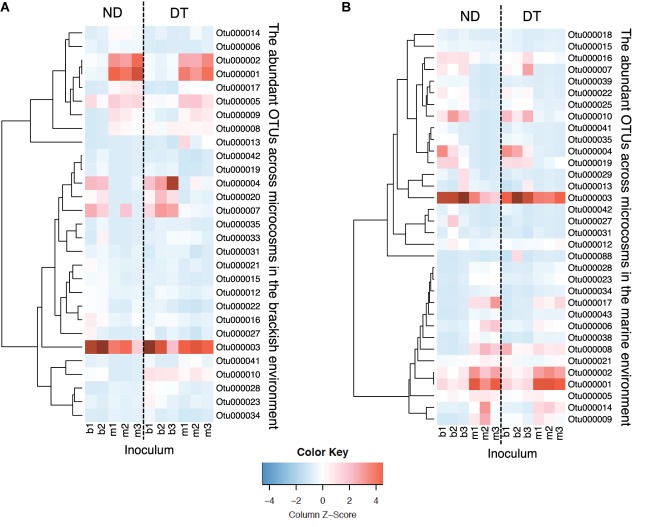
Heatmaps displaying relative abundances of the abundant OTUs (rows) across microcosms (columns) in the brackish **(A)** and in the marine **(B)** environments. Color gradients represent the relative abundances of individual OTUs by column, with warm colors (toward red) indicating abundant OTUs and cold colors (toward blue) indicating rare OTUs within that sample. Column labels are sample IDs (the terminal number represents the biological replicate), and row labels the OTU IDs. The taxonomic affiliation of each of the abundant OTUs is provided in the **Supplementary Table S6**. Dashed-lines in the heatmaps separate between ND (non-dispersal) and DT (dispersal) treatments. Side dendrograms cluster OTUs that have similar occurrence patterns.

Among the 34 abundant OTUs identified across all microcosms, 21 had their maximal abundance in the brackish and 13 in the marine communities (**Supplementary Table S6**). An analysis of the occurrence patterns of the abundant OTUs showed that regardless of the dispersal manipulation, the fraction of abundant OTUs was higher in the transplanted brackish than in the transplanted marine communities (**Figure 6**). Among the abundant OTU pool, Otu000003, affiliated with the family *Campylobacteraceae*, was the most prevalent across microcosms, with a maximal relative abundance of 19.85% in the Bb_ND microcosm (**Figure 6** and **Supplementary Table S6**). Furthermore, several abundant OTUs defined as abundant dispersers in the DT microcosms were rare in the ND microcosms, for either brackish or marine communities (**Figure 6** and **Table 1**). The fraction of the abundant dispersers represented 31% and 14% of the total number of abundant OTUs (across microcosms) in the brackish and marine environments, respectively (**Table 1** and **Figure 6**). Among the OTUs identified as abundant dispersers, Otu000008, affiliated with the family *Rhodobacteraceae*, and Otu000009, affiliated with the family *Colwelliaceae*, showed good dispersal capabilities in the brackish communities, irrespective of the environment. Conversely, the dispersal capacity of Otu000010, affiliated with the family *Bdellovibrionaceae* was good in the marine communities (**Table 1** and **Figure 6**).

**Table 1:** The abundant dispersers in the brackish and marine communities of each environment.

OTU ID	Brackish environment		Marine environment		Taxonomic assignment
	Dispersal capability	Relative abundance %	Dispersal capability	Relative abundance %	
Otu000004	m	1.99%	–	–	*Proteobacteria; Gammaproteobacteria*; unclassified; unclassified; unclassified
Otu000006	–	–	b	1.15%	*Proteobacteria; Gammaproteobacteria; Oceanospirillales; Oceanospirillaceae; Pseudospirillum*
Otu000008	b	3.12%	b	4.19%	*Proteobacteria; Alphaproteobacteria; Rhodobacterales; Rhodobacteraceae; Ascidiaceihabitans*
Otu000009	b	1.24%	b	1.75%	*Proteobacteria; Gammaproteobacteria; Alteromonadales; Colwelliaceae; Colwellia*
Otu000010	m	3.69%	m	1.13%	*Proteobacteria; Deltaproteobacteria; Bdellovibrionales; Bdellovibrionaceae; OM27 clade*
Otu000012	m	1.32%	–	–	*Proteobacteria; Alphaproteobacteria; Rhodobacterales; Rhodobacteraceae; Planktomarina*
Otu000019	–	–	m	1.42%	*Proteobacteria; Gammaproteobacteria; Alteromonadales; Colwelliaceae; Thalassotalea*
Otu000023	b/m	2.46%/1.14%	–	–	*Bacteroidetes; Flavobacteriia; Flavobacteriales; Flavobacteriaceae; Polaribacter*
Otu000028	b/m	2.74%/1.11%	–	–	*Bacteroidetes; Flavobacteriia; Flavobacteriales; Flavobacteriaceae; Dokdonia*
Otu000033	m	1.70%	–	–	*Proteobacteria; Deltaproteobacteria; Desulfuromonadales; GR-WP33-58;* unclassified
Otu000041	m	2.05%	–	–	*Proteobacteria; Deltaproteobacteria; Desulfuromonadales; GR-WP33-58;* unclassified

*Abbreviations ‘b’ and ‘m’ represent the brackish and marine communities in which abundant OTUs showed a potentially high dispersal capability, respectively. For example, the identification of the abundant OTUs in one of the environments as the ‘abundant dispersers’ in the brackish communities implies that these OTUs were detected in the pool of abundant OTUs in the dispersal treatments, but were absent from the brackish communities in the non-dispersal treatments. ‘–’ indicates that the OTUs were not identified as abundant dispersers, that is, they were either rare in a particular incubation environment (mean relative abundance <1% in any microcosm) or abundant in all ND for that environment. Relative abundances are presented as the averages of the replicate samples.*

## Discussion

In this study, we experimentally tested the dispersal dependence of the responses of estuarine bacterioplankton communities exposed to a salinity disturbance (i.e., a difference in salinity between treatments). Our first hypothesis, that dispersal increases the diversity of the communities experiencing a disturbance, was supported only in the case of the marine, not the brackish community. In the marine community, both the realized richness and the evenness increased due to dispersal; this was surprising because in a previous simulation model similar patterns were observed only at high dispersal rates (at least 25%), as a consequence of mass effects (Evans et al., 2017). In our study, mass effects were unlikely to have played a role in our experiment because DT microcosms were subjected to a daily exchange of only 6% (v/v), which is lower than the rates at which mass effects have been shown to occur (Lindström and Östman, 2011; Souffreau et al., 2014). Instead, the frequent dispersal (exchange of cells twice per day) of the brackish into the marine community was enough to allow different taxa to colonize niche space that the marine community alone could not fill. Nonetheless, the dispersal rate was not high enough to result in very high densities of individuals, thereby eliminating the dominant species in the receiving community. Contrary to our hypothesis, however, dispersal did not increase the alpha diversity of brackish communities exposed to the marine environment. This might be a result of the significantly higher initial diversity of the brackish compared to the marine inoculum, since it has been shown that effects of dispersal may depend on initial diversity (Roy et al., 2013; Zha et al., 2016) and that communities with high initial diversity are less susceptible to the dispersing community. To summarize, our results indicate that dispersal maintains or increases alpha community diversity, with the final outcome depending on the initial diversity of the active communities.

As expected, brackish and marine communities became more similar in the presence of dispersal, regardless of the incubation environment (**Figure 4**), which is in agreement with previous studies (Lindström and Östman, 2011; Declerck et al., 2013; Severin et al., 2013). Although the inoculum source and incubation environment explained a large share of the variation in community composition, it was also significantly predicted by dispersal and its interactions with these two other factors (**Supplementary Table S3A**). We also observed that, without dispersal, the relative abundance of rare taxa originating from the marine communities increased in response to a salinity change, while the majority of the taxa from the brackish communities remained abundant. These patterns suggested the existence of a “seed bank” of brackish bacteria within the marine species pool and the ability of some abundant members in the brackish community to readily grow in the marine environment. Similar findings were obtained in a transplant experiment using bacterial assemblages inhabiting the Baltic Sea (Shen et al., 2018).

In aquatic microbial communities, dispersal occurs as community coalescence, i.e., involves not only the mixing of cells between communities but also their respective environmental matrices (Rillig et al., 2015; Mansour et al., 2018). A reduction in beta diversity in response to varying dispersal levels may thus also result from the homogenization of environmental conditions that often accompanies the passive migration of aquatic communities (Lindström et al., 2006; Adams et al., 2014; but see Declerck et al., 2013, where this is described to occur at a very low rate). In our study, the dispersal manipulation was started after the salinity within the dialysis bags and incubation tank had equalized so that confounding environmental effects would be minimized. We found that all measured environmental parameters were similar in the microcosms that were exposed to the same incubation environment at the end of the experiment, irrespective of the origin of the microbes (**Supplementary Figure S1**), which suggests that dissolved nutrients and DOC diffused efficiently through dialysis membranes during the experiment. This is further supported by the finding that the DT treatments in either brackish or marine environment grouped more closely together with respect to similarity of community composition (**Figure 4A**) than to that of the experimental conditions (**Supplementary Figure S1**), e.g., Bb_DT vs. Bm_DT, correspondingly. Therefore, environmental mixing as a confounding factor was minimized in our experiment and the changes in the active bacterial community composition introduced by dispersal were primarily due to the exchange of microorganisms. However, the possibility that the concentrations of DOC and other nutrients in the dialysis bags and incubation tank at the onset of dispersal were not yet in equilibrium, thereby leading to a short-term transient effect of changes in DOC and nutrients, cannot be excluded.

The compositional resemblance following dispersal was more pronounced in our study than in earlier studies, including those with similar dispersal rates (Lindström and Östman, 2011; Severin et al., 2013; Souffreau et al., 2014). This can perhaps be explained by the greater response to dispersal of the metabolically active community examined in our study (based on RNA) than of the total bacterial community (based on DNA) analyzed in those previous studies, as changes in RNA occur faster than changes in DNA (De Vrieze et al., 2016).

There are two potential explanations for the increases in community similarity in response to dispersal, as observed based on assessment of the abundant OTU pool: replacement of OTUs and a change in relative OTU abundance. In the former case, the brackish and marine communities in DT would have shared a large fraction of the abundant OTUs, whereas these would have been rare or absent in the ND. However, this was not the case because only a small fraction of the abundant dispersers (14% and 31% relative to the total number of the abundant OTUs for marine and brackish environments, respectively) was rare in ND. Instead, the relative abundances of the majority of the abundant OTUs changed following dispersal, with the differences in their relative abundances between brackish and marine communities became smaller in DT treatment than ND. These patterns were particularly strong for OTUs originating from the marine inoculum (**Figure 6** and **Supplementary Table S3B**). Hence, our results suggest that increases in community similarity as a response to dispersal are mainly due to changes in relative abundances rather than to replacement of the abundant OTUs.

In addition to the compositional changes at the OTU-level, variation in the relative abundances occurred at higher levels of phylogenetic resolution, particularly at the family level, in response to dispersal. This generally supports our third hypothesis that the importance of dispersal differs among bacterial phylogenetic groups. For example, dispersal promoted the growth of *Bdellovibrionaceae*, a group of prokaryotic bacteriovores (Pineiro et al., 2004). These bacteria were rare and/or dormant in the initial marine inocula and in the marine communities at the end of the experiment without dispersal. The transition of this family from dormancy to active growth was most likely because dispersal substantially influenced prey-predator dynamics (Otto et al., 2017).

Several lines of evidence in this study point to an interaction of dispersal with the incubation environment to influence community assembly. This interaction was more apparent in the brackish than in the marine environment. Evans et al. (2017) proposed that strongly selecting conditions could lead to a small community size (total number of individuals) that is more susceptible to dispersal. However, this was unlikely to have been the case in our study, because the environment had significant effects on the total bacterial densities and higher cell abundances were observed under brackish conditions. Alternatively, the metabolic plasticity of brackish bacteria to salinity disturbances may hinder colonization by an external source, i.e., a marine species pool, thereby decoupling the effect of dispersal from that of saline conditions. In our study, the community memberships of the active brackish communities did not vary substantially between two incubation conditions (Bb_ND vs. Mb_ND; **Figure 5B**). In addition, after the exposure of those communities to dispersal in the marine environment (Mb_DT vs. Mm_DT), their composition was less similar to the marine communities than to those of the brackish environment (Bb_DT vs. Bm_DT; **Figure 5B**). This hypothesis should be tested in further studies examining how dispersal modifies the response of brackish bacteria to a wide range of salinities.

A few concerns need to be addressed when experimental findings are extrapolated to natural systems. First, the patterns observed in this study were derived from two sources of microbial inoculum. This limited dispersal source may have missed functionally rich communities and/or rich pools of potential immigrants (Comte et al., 2017), both of which could have fueled natural metacommunity dynamics. Second, microcosms are regarded as artificial systems, in which either the resulting communities differ from the composition of the original inoculum or where fast-growing opportunists are simply enriched (Christian and Capone, 2002; Aanderud et al., 2015). For instance, Otu000003 affiliated with the genus of *Arcobacter* was prevalent in our microcosms (**Supplementary Table S6**); this opportunistic taxa may benefit from resource competition and shifts in nutrient availability during the incubation. Nevertheless, as the communities originating from the same inoculum still clustered together at the end of the experiment (Bb vs. Mb; Bm vs. Mm; **Figure 4A**), the observed compositional differences can most likely be attributed to dispersal. More importantly, our aim was to assess the direct dispersal effects imposed by the passive transport of the cells on the response of communities to new environmental conditions, which could only be accomplished in a microcosm-type system.

In summary, we have shown that a relatively low rate of exchange of microorganisms among local communities can alter the importance of environmental effects induced by disturbances on the active bacterial communities. This response was likely driven by changes in the relative abundances rather than by a major replacement of abundant taxa. Moreover, the interactive effects of dispersal and contemporary environmental conditions were stronger at lower taxonomic levels, which facilitated the local adjustment of some bacteria in brackish waters. Our study provides a better understanding of the fate of dispersed bacteria under saltwater inflow scenarios and enhances efforts at predicting bacterial responses to environmental changes.

## Author Contributions

DS and KJ designed the experiments. DS performed the experiments, processed the samples, analyzed the data, and wrote the manuscript. SL and KJ provided guidance in writing and commented on earlier drafts. All authors discussed and approved the manuscript.

## Conflict of Interest Statement

The authors declare that the research was conducted in the absence of any commercial or financial relationships that could be construed as a potential conflict of interest.

## Footnotes

1 https://www.mothur.org/wiki/MiSeq_SOP

2 http://www.ebi.ac.uk/ena

## References

[B1] Aanderud Z. Y. Jones S. E. Fierer N. Lennon J. T. (2015). Resuscitation of the rare biosphere contributes to pulses of ecosystem activity. *Front. Microbiol.* 6:24. 10.3389/fmicb.2015.00024 25688238 PMC4311709

[B2] Adams H. E. Crump B. Kling G. W. (2014). Metacommunity dynamics of bacteria in an artic lake: the impact of species sorting and mass effects on bacterial production and biogeography. *Front. Microbiol.* 5:82 10.3389/fmicb.2014.00082 PMC3940886 24624127

[B3] Anderson M. J. (2001). Permutation tests for univariate or multivariate analysis of variance and regression. *Can. J. Fish. Aquat. Sci.* 58 626–639. 10.1139/f01-004

[B4] Anderson M. J. (2006). Distance-based tests for homogeneity of multivariate dispersions. *Biometrics* 62 245–253. 10.1111/j.1541-0420.2005.00440.x 16542252

[B5] Baho D. L. Peter H. Tranvik L. J. (2012). Resistance and resilience of microbial commuities – temporal and spatial insurance against perturbations. *Environ. Microbiol.* 14 2283–2292. 10.1111/j.1462-2920.2012.02754.x 22513226

[B6] Berga M. Östman Ö Lindström E. S. Langenheder S. (2015). Combined effects of zooplankton grazing and dispersal on the diversity and assembly mechanisms of bacterial metacommunities. *Environ. Microbiol.* 17 2275–2287. 10.1111/1462-2920.12688 25367396

[B7] Blazewicz S. J. Barnard R. L. Daly R. A. Fireston M. K. (2013). Evaluating rRNA as an indicator of microbial activity in environmental communities: limitations and uses. *ISME J.* 7 2061–2068. 10.1038/ismej.2013.102 23823491 PMC3806256

[B8] Bouvier T. C. del Giorgio P. A. (2002). Compositional changes in free-living bacterial communities along a salinity gradient in two temperate estuaries. *Limnol. Oceanogr.* 47 453–470. 10.4319/lo.2002.47.2.0453

[B9] Chesson P. (2000). Mechanisms of maintenance of species diversity. *Annu. Rev. Ecol. Syst.* 31 343–366. 10.1146/annurev.ecolsys.31.1.343

[B10] Christian R. R. Capone D. G. (2002). “Overview of issues in aquatic microbial ecology,” in *Manual of Environmental Microbiology*, 2nd Edn, eds Hurst C. J. Crawford R. L. Kundsen G. R. Mclnerney M. J. Stetzenbach L. D. (Washington, DC: ASM Press), 323–328.

[B11] Comte J. Langenheder S. Berga M. Lindstöm E. S. (2017). Contribution of different dispersal sources to the metabolic response of lake bacterioplankton following a salinity change. *Environ. Microbiol.* 19 215–260. 10.1111/1462-2920.13593 27871136

[B12] De Vrieze J. D. Regueiro L. Props R. Vilchez-Vargas R. Jáuregui R. Pieper D. H. (2016). Presence does not imply activity: DNA and RNA patterns differ in response to salt pertubation in anaerobic digestion. *Biotechnol. Biofuels* 9:244. 10.1186/s13068-016-0652-5 27843490 PMC5103597

[B13] Declerck S. A. Winter C. Shurin J. B. Suttle C. A. Matthews B. (2013). Effects of patch connectivity and heterogeneity on metacommunity structure of planktonic bacteria and viruses. *ISME J.* 7 533–542. 10.1038/ismej.2012.138 23178674 PMC3578570

[B14] Dinauer A. Mucci A. (2017). Spatial variability in surface-water pCO2 and gas exchange in the world’s largest semi-enclosed estuarine system: St. Lawrence Estuary (Canada). *Biogeosciences* 14 3221–3237. 10.5194/bg-14-3221-2017

[B15] Edgar R. C. Haas B. J. Clemente J. C. Quince C. Knight R. (2011). UCHIME improves sensitivity and speed of chimera detection. *Bioinformatics* 27 2194–2200. 10.1093/bioinformatics/btr381 21700674 PMC3150044

[B16] Evans S. Martiny J. B. H. Allison S. D. (2017). Effects of dispersal and selection on stochastic assembly in microbial communities. *ISME J.* 11 176–185. 10.1038/ismej.2016.96 27494293 PMC5315486

[B17] Gasol J. M. del Giorgio P. A. (2000). Using flow cytometry for counting natural planktonic bacteria and understanding the structure of planktonic bacterial communities. *Sci. Mar.* 64 197–224. 10.3989/scimar.2000.64n2197

[B18] Grasshoff K. Kremling K. Ehrhardt M. (eds). (1999). *Methods of Seawater Analysis*, 3rd Edn. Weinheim: Wiley-VCH Press, 159–228. 10.1002/9783527613984

[B19] Herbert E. R. Boon P. Burgin A. J. Neubauer S. C. Franklin R. B. Ardón M. (2015). A global perspective on wetland salinization: ecological consequences of a growing threat to freshwater wetlands. *Ecosphere* 6 1–43. 10.1890/ES14-00534.1

[B20] Herlemann D. P. Labrenz M. Jürgens K. Bertilsson S. Waniek J. J. Andersson A. F. (2011). Transitions in bacterial communities along the 2000 km salinity gradient of the Baltic Sea. *ISME J.* 5 1571–1579. 10.1038/ismej.2011.41 21472016 PMC3176514

[B21] Jones S. E. Lennon J. T. (2010). Dormancy contributes to the maintenance of microbial diversity. *Proc. Natl. Acad. Sci. U.S.A.* 107 5881–5886. 10.1073/pnas.0912765107 20231463 PMC2851880

[B22] Keer J. T. Birch L. (2003). Molecular methods for the assessment of bacterial viability. *J. Microbiol. Methods* 53 175–183. 10.1016/S0167-7012(03)00025-3 12654489

[B23] Kozich J. J. Westcott S. L. Baxter N. T. Highlander S. K. Schloss P. D. (2013). Development of a dual-index sequencing strategy and curation pipeline for analyzing amplicon sequence data on the MiSeq Illumina sequencing platform. *Appl. Environ. Microbiol.* 79 5112–5120. 10.1128/AEM.01043-13 23793624 PMC3753973

[B24] Leibold M. A. Holyoak M. Mouquet N. Amarasekare P. Chase J. M. Hoopes M. F. (2004). The metacommunity concept: a framework for multiscale community ecology. *Ecol. Lett.* 7 601–613. 10.1111/j.1461-0248.2004.00608.x

[B25] Lindström E. S. Forslund M. Algesten G. Bergström A. (2006). External control of bacterial community sturcture in lakes. *Limnol. Oceanogr.* 51 339–342. 10.4319/lo.2006.51.1.0339

[B26] Lindström E. S. Langenheder S. (2012). Local and regional factors influencing bacterial community assembly. *Environ. Microbiol. Rep.* 4 1–9. 10.1111/j.1758-2229.2011.00257.x 23757223

[B27] Lindström E. S. Östman Ö (2011). The importance of dispersal for bacterial community composition and functioning. *PLoS One* 6:e25883. 10.1371/journal.pone.0025883 21998714 PMC3188564

[B28] Livingston G. Matias M. Calcagno V. Barbera C. Combe M. Leibold M. A. (2012). Competition-colonization dynamics in experimental bacterial metacommunities. *Nat. Commun.* 3:1234. 10.1038/ncomms2239 23212363

[B29] Loreau M. Mouquet N. (1999). Immigration and the maintenance of local species diversity. *Am. Nat.* 154 427–440. 10.1086/303252 10523489

[B30] Lowe W. H. McPeek M. A. (2014). Is dispersal neutral? *Trends Ecol. Evol.* 29 444–450. 10.1016/j.tree.2014.05.009 24962790

[B31] Lozupone C. A. Knight R. (2007). Global patterns in bacterial diversity. *Proc. Natl. Acad. Sci. U.S.A.* 104 11436–11440. 10.1073/pnas.0611525104 17592124 PMC2040916

[B32] Mansour I. Heppell C. M. Ryo M. Rillig M. C. (2018). Application of the microbial community coalescence concept to riverine networks. *Biol. Rev.* 10.1111/brv.12422 [Epub ahead of print]. 29700966

[B33] Martiny J. B. Bohannan B. J. Brown J. H. Colwell R. K. Fuhrman J. A. Green J. L. (2006). Microbial biogeography: putting microorganisms on the map. *Nat. Rev. Microbiol.* 4 102–112. 10.1038/nrmicro1341 16415926

[B34] Martiny J. B. H. (2015). Dispersal and the microbiome. *Microbe* 10 191–196. 10.1128/microbe.10.191.1

[B35] Matthiessen B. Hillebrand H. (2006). Dispersal frequency affects local biomass production by controlling local diversity. *Ecol. Lett.* 9 652–662. 10.1111/j.1461-0248.2006.00916.x 16706910

[B36] Mohrholz V. Naumann M. Nausch G. Krüger S. Gräwe U. (2015). Fresh oxygen for the Baltic Sea – An exceptional saline inflow after a decade of stagnation. *J Mar. Syst.* 148 152–166. 10.1016/j.jmarsys.2015.03.005

[B37] Mouquet N. Loreau M. (2003). Community patterns in source-sink metacommunities. *Am. Nat.* 162 544–557. 10.1086/378857 14618534

[B38] Mucci A. Starr M. Gilbert D. Sundby B. (2011). Acidification of lower St. Lawrence Estuary bottom waters. *Atmos. Ocean* 49 206–218. 10.1080/07055900.2011.599265

[B39] Nebbioso A. Piccolo A. (2013). Molecular characterization of dissolved organic matter (DOM): a critical review. *Anal. Bioanal. Chem.* 405 1–16. 10.1007/s00216-012-6363-2 22965531

[B40] Nemergut D. R. Schmidt S. K. Fukami T. O’Neill S. P. Bilinski T. M. Stanish L. F. (2013). Patterns and processes of microbial community assembly. *Microbiol. Mol. Biol. Rev.* 77 342–356. 10.1128/MMBR.00051-12 24006468 PMC3811611

[B41] Nielsen K. M. Johnsen P. J. Bensasson D. Daffonchio D. (2007). Release and persistence of extracellular DNA in the environment. *Environ. Biosafety Res.* 6 37–53. 10.1051/ebr:2007031 17961479

[B42] Oksanen J. Blanchet F. G. Friendly M. Kindt R. Legendre P. McGlinn D. (2016). *Paclage ‘Vegan’: Community Ecology Package*. Available at: https://cran.r-project.org/web/packages/vegan/vegan.pdf

[B43] Otto S. Bruni E. P. Harms H. Wick L. Y. (2017). Catch me if you can: dispersal network and foraging of *Bdellovibrio bacteriovorus* 109J along mycelia. *ISME J.* 11 386–393. 10.1038/ismej.2016.135 27824344 PMC5270576

[B44] Pineiro S. A. Sahaniuk G. E. Romberg E. Williams H. N. (2004). Predation pattern and phylogenetic analysis of *Bdellovibrionaceae* from the Great Salt lake. Utah. *Curr. Microbiol.* 48 113–117. 10.1007/s00284-003-4136-z 15057478

[B45] Rillig M. C. Antonovics J. Caruso T. Lehmann A. Powell J. R. Veresoglou S. D. (2015). Interchange of entire communities: microbial community coalescence. *Trends Ecol. Evol.* 30 470–476. 10.1016/j.tree.2016.06.004 26111582

[B46] Roy K. D. Marzorati M. Negroni A. Thas O. Balloi A. Fabio F. (2013). Environmental conditions and community evenness determine the outcome of biological invasion. *Nat. Commun.* 4:1383. 10.1038/ncomms2392 23340423

[B47] Severin I. Östman Ö Lindström E. S. (2013). Variable effects of dispersal on productivity of bacterial communities due to changes in functional trait composition. *PLoS One* 8:e80825. 10.1371/journal.pone.0080825 24324633 PMC3851979

[B48] Shade A. Peter H. Allison S. D. Baho D. L. Berga M. Bürgmann H. (2012). Fundamentals of microbial community resistance and resilience. *Front. Microbiol.* 3:417. 10.3389/fmicb.2012.00417 23267351 PMC3525951

[B49] Shen D. Jürgens K. Beier S. (2018). Experimental insights into the importance of ecologically dissimilar bacteria to community assembly along a salinity gradient. *Environ. Microbiol.* 20 1170–1184. 10.1111/1462-2920 29393568

[B50] Souffreau C. Pecceu B. Denis C. Rummens K. Meester L. D. (2014). An experimental analysis of species sorting and mass effects in freshwater bacterioplankton. *Freshw. Biol.* 59 2081–2095. 10.1111/fwb.12408

[B51] Stolle C. Labrenz M. Meeske C. Jürgens K. (2011). Bacterioneuston community structure in the southern Baltic Sea and its dependence on meteorological conditions. *Appl. Environ. Microbiol.* 77 3726–3733. 10.1128/AEM.00042-11 21478321 PMC3127628

[B52] Székely A. J. Berga M. Langenheder S. (2013). Mechanisms determining the fate of dispersed bacterial communities in new environments. *ISME J.* 7 61–71. 10.1038/ismej.2012.80 22810061 PMC3526183

[B53] Székely A. J. Langenheder S. (2017). Dispersal timing and drought history influence the response of bacterioplankton to drying – rewetting stress. *ISME J.* 11 1764–1776. 10.1038/ismej.2017.55 28440801 PMC5520042

[B54] Vuono D. C. Munakata-Marr J. Spear J. R. Drewes J. E. (2016). Disturbance opens recruitment sites for bacterial colonization in activated sludge. *Environ. Microbiol.* 18 87–99. 10.1111/1462-2920.12824 25727891

[B55] Wang Q. Garrity G. M. Tiedje J. M. Cole J. R. (2007). Naive Bayesian classifier for rapid assignment of rRNA sequences into the new bacterial taxonomy. *Appl. Environ. Microbiol.* 73 5261–5267. 10.1128/AEM.00062-07 17586664 PMC1950982

[B56] Weber F. Campo J. Wylezich C. Massana R. Jürgens K. (2012). Unveiling trophic functions of uncultured protist taxa by incubation experiments in the brackish Baltic Sea. *PLoS One* 7:e41970. 10.1371/journal.pone.0041970 22860041 PMC3408427

[B57] Wu F. C. Tanoue E. Liu C. Q. (2003). Fluorescence and amino acid characteristics of molecular size fractions of DOM in the waters of Lake Biwa. *Biogeochemistry* 65 245–257. 10.1023/A:1026074318377

[B58] Zha Y. Berga M. Comte J. Langenheder S. (2016). Effects of dispersal and initial diversity on the composition and functional performance of bacterial communities. *PLoS One* 11:e0155239. 10.1371/journal.pone.0155239 27182596 PMC4868275

