# Supplementary material for: Dispersal Modifies the Diversity and Composition of Active Bacterial Communities in Response to a Salinity Disturbance
Source: Front Microbiol. 2018 Sep 19;9:2188. doi: 10.3389/fmicb.2018.02188 (PMC6159742; doi:10.3389/fmicb.2018.02188)
Supplement: Supplementary file 1 [file Data_Sheet_1.PDF]

Supplemental materials for

Dispersal modifies the diversity and composition of active bacterial communities in response to a salinity disturbance

Dandan Shen[1*], Silke Langenheder[2], Klaus Jürgens[1]

[1] *Leibniz Institute for Baltic Sea Research Warnemünde (IOW), Biological Oceanography, Seestr. 15, D- 18119 Rostock, Germany*

[2] *Department of Ecology and Genetic/Limnology, Evolutionary Biology Centre, Uppsala University, Norbyvägen 18 D, 75236, Uppsala, Sweden*

*Correspondence
Dandan Shen,
dand.shen@gmail.com

Contents

Supplemental Figure S1

Supplemental Table S1

Supplemental Table S2

Supplemental Table S3

Supplemental Table S4

Supplemental Table S5

Supplemental Table S6

Figure S1 Principal component analysis (PCA) based on the measured salinity, nutrient concentrations, DOC and PA in the samples at the end of the experiment (day 5). The particular combination of incubation environment and inoculum source is color-coded: b inoculum in the B environment is (Bb: orange), m inoculum in the B environment (Bm: olivegreen), b inoculum in the M environment (Mb: brown), and m inoculum in the M environment (Mm: dark green). ND: non-dispersal treatment (×), DT: dispersal treatment (triangles). The values obtained from the measurements of NO_2^- and NH_4^+ were below detectable lilmits and thus were excluded from the analysis. The two first axes explained 86% of the variance. Abbreviation: [DOC] Dissolved organic carbon, [PA] Protist abundance.

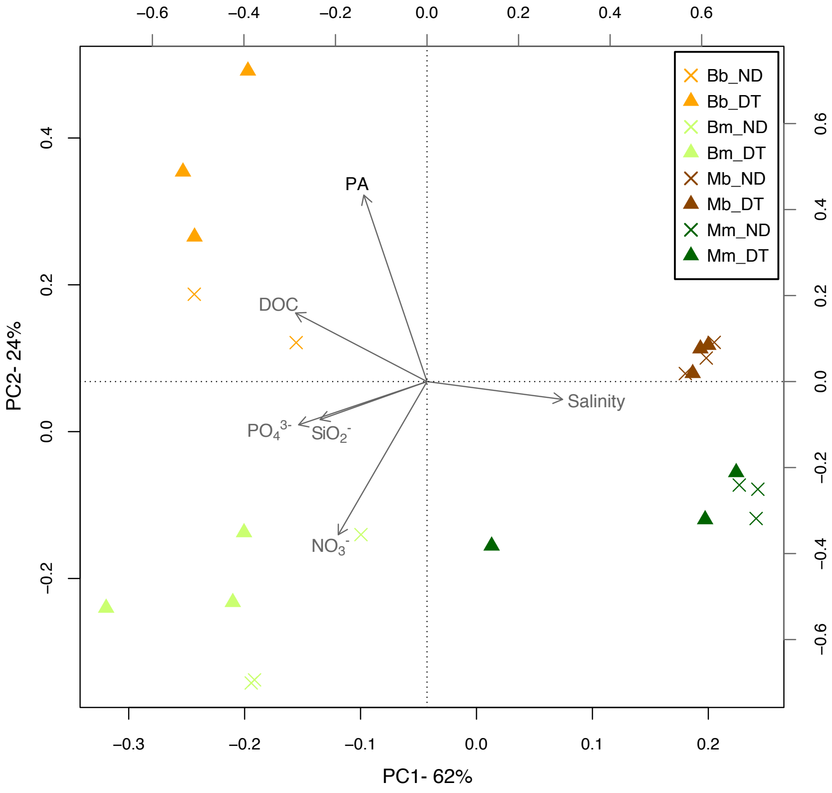

Table legends

Table S1 Nutrient content, microbial cell abundances, number of sequence reads and bacterial community diversity metrics in each biological replicate of the initial inocula (day 0) and in the microcosms at the end of the expeirment (day 5). Capital letters B and M refer to the incubation environment, and lower case letters b and m refer to the source (origin) of the initial microbial inoculum. ND and DT indicate non-dispersal and dispersal treatments, respectively; the terminal number represents the biological replicate. Abbreviations: psu: practical salinity unit; DOC: dissolved organic carbon. NA: no available data.

**Sample ID**	**Salinity** (psu)	**NO_3^-** (μM)	**NO_2^-** (μM)	**PO_4^{3-}** (μM)	**NH_4^+** (μM)	**SiO_2^-** (μM)	**DOC** (μM)	**Bacteria** x 10^6 (cells ml^{-1})	**Protists** x 10^3 (cells ml^{-1})	**No. reads**	**Shannon diversity**	**Richness**	**Evenness**
Initial_b1	24.29	11.71	0.19	0.87	0.10	16.91	157.60	0.87	1.85	22544	4.52	590.24	0.64
Initial_b2	NA	NA	NA	NA	NA	NA	NA	0.98	1.62	23646	4.59	612.04	0.65
Initial_b3	NA	NA	NA	NA	NA	NA	NA	1.04	1.48	29380	4.63	630.03	0.65
Initial_m1	30.35	1.09	0.07	0.03	0.10	1.84	107.00	0.87	2.86	23552	3.57	323.25	0.55
Initial_m2	NA	NA	NA	NA	NA	NA	NA	0.87	3.76	28499	3.49	313.35	0.54
Initial_m3	NA	NA	NA	NA	NA	NA	NA	0.90	3.68	22369	3.47	320.52	0.54
Bb_ND1	24.59	1.33	0.09	0.3	0.1	13.21	154.05	1.91	12.08	36177	3.76	502.20	0.54
Bb_ND2	25	0.98	0.01	0.26	0.1	12.49	148.05	1.90	7.08	11877	3.56	391.00	0.53
Bm_ND1	24.59	1.05	0.06	0.39	0.1	12.41	149.2	3.09	1.87	25925	3.24	456.67	0.48
Bm_ND2	25	0.78	0.05	0.2	0.1	12.49	149.9	3.94	1.89	12980	3.19	397.69	0.48
Bm_ND3	24.78	1.12	0.06	0.35	0.1	12.79	149.25	3.53	1.66	19957	3.31	548.31	0.47
Bb_DT1	25.28	2.78	0.01	0.28	0.1	12.62	135.3	1.83	14.60	22494	3.85	582.87	0.55
Bb_DT2	25.2	1.56	0.01	0.24	0.1	12.56	131.2	2.21	21.22	28511	3.71	571.91	0.53
Bb_DT3	25.2	2.68	0.01	0.33	0.1	12.84	132.8	1.51	18.82	32284	3.95	686.88	0.55

Sample ID	Salinity (psu)	NO_3^- (µM)	NO_2^- (µM)	PO_4^{3-} (µM)	NH_4^+ (µM)	SiO_2^- (µM)	DOC (µM)	Bacteria x 10^6 (cells ml^{-1})	Protists x 10^3 (cells ml^{-1})	No. reads	Shannon diversity	Richness	Evenness
Bm_DT1	25.28	2.09	0.07	0.67	0.1	15.47	144.3	3.11	3.33	13594	3.88	612.56	0.55
Bm_DT2	25.2	2.07	0.09	0.44	0.1	13.37	136.3	3.24	2.49	37647	3.91	576.24	0.55
Bm_DT3	25.2	1.71	0.06	0.5	0.1	12.7	132.5	2.71	4.84	26026	3.78	739.64	0.52
Mb_ND1	29.32	0.68	0.01	0.03	0.1	3.28	117.85	0.40	5.48	15761	4.16	744.92	0.57
Mb_ND2	29.07	0.62	0.01	0.03	0.1	3.26	113.7	0.40	6.45	14950	4.01	644.43	0.56
Mb_ND3	29.27	0.69	0.01	0.11	0.1	3.32	115.85	0.48	5.45	41150	3.89	723.31	0.53
Mm_ND1	29.34	0.64	0.01	0.11	0.1	3.32	115.7	2.52	1.46	11074	3.68	445.60	0.54
Mm_ND2	29.27	0.64	0.01	0.03	0.1	3.1	117.35	2.19	1.00	39650	3.55	412.91	0.53
Mm_ND3	29.27	0.6	0.01	0.03	0.1	3.13	121.45	2.31	1.28	16192	3.40	316.18	0.53
Mb_DT1	29.45	1.01	0.01	0.03	0.1	3.58	109.33	0.59	5.14	19803	4.15	641.48	0.58
Mb_DT2	29.78	0.96	0.01	0.03	0.1	3.46	108.55	0.75	5.98	19990	4.04	719.85	0.56
Mb_DT3	29.78	1.18	0.01	0.03	0.1	3.36	105.05	0.61	4.96	21006	4.04	690.55	0.56
Mm_DT1	30.1	1.08	0.1	0.13	0.1	27.06	118	2.05	2.46	18370	3.66	537.54	0.52
Mm_DT2	29.75	1.16	0.01	0.11	0.1	5.96	104.95	2.58	2.14	29062	3.40	451.97	0.50
Mm_DT3	30.1	1.01	0.01	0.03	0.1	3.75	113.95	2.31	1.81	21095	3.52	393.69	0.53

The detection limit for colorimetric nutrient measurements was: NO_3^-, 0.05 µM; NO_2^-, 0.01 µM; PO_4^{3-}, 0.03 µM; NH_4^+, 0.1 µM, after Strickland and Parsons (1972).

Reference

Strickland, J.D.H., and Parsons, T.R. (1972). A Practical Handbook of Seawater Analysis, 2nd Edition (Fisheries Research Board of Canada, Bulletin No. 167).

Table S2 The results of three-way ANOVAs testing the effects of dispersal level, incubation environment, inoculum source and their interactions on microbial abundance and bacterial community diversity.

	**Bacterial abundance**		**Protist abundance**		**Shannon diversity**		**Richness**		**Evenness**	
Factors	F	*P*-value	F	*P*-value	F	*P*-value	F	*P*-value	F	*P*-value
**Dispersal**	0.93	ns	40.88	***	20.42	***	10.80	**	8.38	*
**Incubation environment (IncE)**	128.78	***	45.71	***	10.35	**	0.21	ns	13.91	**
**Inoculum source (InoS)**	224.57	***	293.32	***	59.25	***	21.79	***	34.01	***
**Dispersal x IncE**	3.14	**.**	6.85	*	16.53	**	5.39	*	11.68	**
**Dispersal x InoS**	2.90	ns	3.04	ns	3.77	**.**	1.41	ns	2.21	ns
**IncE x InoS**	3.51	**.**	6.55	*	12.33	**	26.05	***	0.21	ns
**Dispersal x IncE x InoS**	0.21	ns	3.64	**.**	6.98	*	0.52	ns	15.22	**

Abbreviation: IncE, incubation environment; InoS, inoculum source; ns, not significant.
Significance codes : '***' $P < 0.001$; '**' $P < 0.01$; '*' $P < 0.05$; '**.**' $P < 0.1$.

Table S3 PERMANOVA tests showing the variance (R^2) explained by dispersal, incubation environment, inoculum source, and their interactions within the community composition among all microcosms (A) and among the microcosms with the brackish vs. marine inoculum sources separately (B).

A.

	Variance (R^2) explained	*P*-value
**Dispersal**	3.50%	*
**Incubation environment (IncE)**	9.32%	**
**Inoculum source (InoS)**	52.23%	***
**Dispesal x IncE**	2.92%	**.**
**Dispersal x InoS**	5.59%	*
**IncE x InoS**	6.62%	**
**Dispesal x IncE x InoS**	1.25%	ns

B.

	**Brackish inoculum source**		**Marine inoculum source**	
	Variance (R^2) explained	*P*-value	Variance (R^2) explained	*P*-value
**Dispersal**	17.66%	*	18.52%	**
**Incubation environment (IncE)**	37.09%	**	31.48%	***
**Dispesal x IncE**	3.82%	ns	14.35%	*

Abbreviation: IncE, incubation environment; InoS, inoculum source; ns, not significant.
Significance codes : '***' $P < 0.001$; '**' $P < 0.01$; '*' $P < 0.05$; '**.**' $P < 0.1$.
Number of permutations: 999

Table S4 Explanatory values of the environmental variables to the differences in community structure along non-metric multidimensional scaling axes for all microcosms. Factors significant at $P < 0.05$ are shown in bold.

	NMDS1	NMDS2	R^2	*P*-value	
**Salinity**	**0.130**	**0.992**	**0.727**	**0.001**	***
**NO_3^-**	**0.432**	**-0.902**	**0.447**	**0.003**	**
NO_2^-	-0.277	-0.961	0.155	0.18	ns
**PO_4^{3-}**	**-0.052**	**-0.999**	**0.496**	**0.002**	***
NH_4^+	0.000	0.000	0.000	1	ns
**SiO_2^-**	**0.039**	**-0.999**	**0.358**	**0.015**	*
**DOC**	**-0.277**	**-0.961**	**0.772**	**0.001**	***
**PA**	**-0.791**	**-0.611**	**0.564**	**0.001**	***

Significance codes : '***' $P < 0.001$; '**' $P < 0.01$; '*' $P < 0.05$; '.' $P < 0.1$.
ns, not significant.
Number of permutations: 999

Table S5 The results of three-way ANOVAs testing the effects of dispersal level, incubation environment, inoculum source and their interactions on the abundances of the main bacterial phyla/classes and their subgroups (bacterial orders and/or families).

A. Alproteobacteria and subgroups

	**Alproteobacteria**		**SAR11 clade**		**Rhodobacteraceae**		**SAR116 clade**	
Factors	F	*P*-value	F	*P*-value	F	*P*-value	F	*P*-value
**Dispersal**	1.43	ns	0.04	ns	0.16	ns	0.10	ns
**Incubation environment (IncE)**	46.43	***	12.14	**	33.34	***	0.60	ns
**Inoculum source (InoS)**	28.72	***	20.91	***	57.99	***	0.01	ns
**Dispersal x IncE**	0.17	ns	3.17	.	0.28	ns	0.42	ns
**Dispersal x InoS**	6.39	*	0.78	ns	10.21	**	0.82	ns
**IncE x InoS**	7.05	*	0.01	ns	6.35	*	1.55	ns
**Dispersal x IncE x InoS**	3.06	ns	5.65	*	1.18	ns	0.06	ns

B. Deltaproteobacteria and subgroup

	**Deltaproteobacteria**		**Bdellovibrionaceae**	
Factors	F	*P*-value	F	*P*-value
**Dispersal**	61.21	***	54.58	***
**Incubation environment (IncE)**	2.88	ns	0.29	ns
**Inoculum source (InoS)**	155.70	***	155.43	***
**Dispersal x IncE**	12.41	**	10.47	**
**Dispersal x InoS**	44.16	***	28.86	***
**IncE x InoS**	16.93	***	24.38	***
**Dispersal x IncE x InoS**	3.13	.	0.00	ns

Abbreviation: IncE, incubation environment; InoS, inoculum source; ns, not significant.
Significance codes : '***' $P < 0.001$; '**' $P < 0.01$; '*' $P < 0.05$; '.' $P < 0.1$.

C. Epsilonproteobacteria and subgroup

	**Epsilonproteobacteria**		**Campylobacteraceae**	
Factors	F	*P*-value	F	*P*-value
**Dispersal**	0.07	ns	0.07	ns
**Incubation environment (IncE)**	2.24	ns	2.23	ns
**Inoculum source (InoS)**	5.44	*	5.46	*
**Dispersal x IncE**	1.73	ns	1.77	ns
**Dispersal x InoS**	5.21	*	5.23	*
**IncE x InoS**	0.03	ns	0.03	ns
**Dispersal x IncE x InoS**	0.08	ns	0.07	ns

D. Gammaproteobacteria and subgroups

	**Gammaproteobacteria**		**Cellvibrionaceae**		**Colwelliaceae**		**Oceanospirillaceae**		**Vibrionaceae**	
Factors	F	*P*-value	F	*P*-value	F	*P*-value	F	*P*-value	F	*P*-value
**Dispersal**	0.43	ns	0.71	ns	0.01	ns	8.78	**	3.32	**.**
**Incubation environment (IncE)**	14.97	**	25.39	***	0.43	ns	1.69	ns	0.07	ns
**Inoculum source (InoS)**	8.68	*	62.29	***	0.05	ns	75.44	***	156.75	***
**Dispersal x IncE**	1.95	ns	0.67	ns	0.05	ns	0.77	ns	8.42	*
**Dispersal x InoS**	0.72	ns	0.01	ns	0.58	ns	1.72	ns	0.92	ns
**IncE x InoS**	1.55	ns	6.51	*	2.53	ns	0.45	ns	5.80	*
**Dispersal x IncE x InoS**	4.70	*	0.28	ns	0.00	ns	1.07	ns	6.35	*

Abbreviation: IncE, incubation environment; InoS, inoculum source; ns, not significant.

Significance codes : '***' $P < 0.001$; '**' $P < 0.01$; '*' $P < 0.05$; '**.**' $P < 0.1$.

E. Bacteroidetes and subgroup

	**Bacteroidetes**		**Flavobacteriaceae**	
Factors	F	*P*-value	F	*P*-value
**Dispersal**	1.53	ns	0.01	ns
**Incubation environment (IncE)**	6.74	*	3.79	.
**Inoculum source (InoS)**	12.68	**	5.06	*
**Dispersal x IncE**	1.73	ns	6.09	*
**Dispersal x InoS**	4.67	*	8.66	*
**IncE x InoS**	23.14	***	30.46	***
**Dispersal x IncE x InoS**	1.83	ns	1.86	ns

Abbreviation: IncE, incubation environment; InoS, inoculum source; ns, not significant.
Significance codes : '***' $P < 0.001$; '**' $P < 0.01$; '*' $P < 0.05$; '.' $P < 0.1$.

Table S6 Taxonomic affiliation and maximal relative abundance of the most abundant OTUs (mean relative abundance > 1% in any microcosm). The numbers in parentheses are the proportion of the sequences for an OTU classified as representing members of the given taxonomy. Abbreviation: Micro_ID, refers to the microcosm in which the maximal relative abundance was detected; Max%, the maximal relative abundance of the given OTU.

OTU ID	Phylum	Class	Order	Family	Genus	Max %	Micro_ID
Otu000001	*Proteobacteria*(100)	*Gammaproteobacteria*(100)	*Vibrionales*(100)	*Vibrionaceae*(100)	Vibrio(95)	18.34	Bm_ND
Otu000002	*Proteobacteria*(100)	*Gammaproteobacteria*(100)	*Vibrionales*(100)	*Vibrionaceae*(100)	unclassified(82)	14.67	Bm_ND
Otu000003	*Proteobacteria*(100)	*Epsilonproteobacteria*(100)	*Campylobacterales*(100)	*Campylobacteraceae*(100)	*Arcobacter*(100)	19.85	Bb_ND
Otu000004	*Proteobacteria*(100)	*Gammaproteobacteria*(100)	unclassified(100)	unclassified(100)	unclassified(100)	11.41	Bb_DT
Otu000005	*Proteobacteria*(100)	*Gammaproteobacteria*(100)	*Oceanospirillales*(100)	*Oceanospirillaceae*(100)	*Marinomonas*(100)	7.64	Bm_ND
Otu000006	*Proteobacteria*(100)	*Gammaproteobacteria*(100)	*Oceanospirillales*(100)	*Oceanospirillaceae*(100)	*Pseudospirillum*(100)	5.17	Mm_ND
Otu000007	*Proteobacteria*(100)	*Gammaproteobacteria*(100)	*Cellvibrionales*(100)	*Cellvibrionaceae*(100)	*Simiduia*(100)	8.57	Bb_DT
Otu000008	*Proteobacteria*(100)	*Alphaproteobacteria*(100)	*Rhodobacterales*(100)	*Rhodobacteraceae*(100)	*Ascidiaceihabitans*(78)	6.42	Mm_ND
Otu000009	*Proteobacteria*(100)	*Gammaproteobacteria*(100)	*Alteromonadales*(100)	*Colwelliaceae*(100)	*Colwellia*(100)	4.87	Bm_ND
Otu000010	*Proteobacteria*(100)	*Deltaproteobacteria*(100)	*Bdellovibrionales*(100)	*Bdellovibrionaceae*(100)	*OM27_clade*(100)	6.56	Mb_ND
Otu000012	*Proteobacteria*(100)	*Alphaproteobacteria*(100)	*Rhodobacterales*(100)	*Rhodobacteraceae*(100)	*Planktomarina*(97)	2.47	Mm_DT
Otu000013	*Proteobacteria*(100)	*Gammaproteobacteria*(100)	*Alteromonadales*(100)	*Pseudoalteromonadaceae*(100)	*Algicola*(100)	3.31	Bm_DT
Otu000014	*Bacteroidetes*(100)	*Cytophagia*(100)	*Cytophagales*(100)	*Flammeovirgaceae*(100)	*Reichenbachiella*(100)	5.85	Mm_DT
Otu000015	*Proteobacteria*(100)	*Alphaproteobacteria*(100)	*Rickettsiales*(100)	*SAR116_clade*(100)	*Candidatus_Puniceispirillum*(100)	2.14	Bb_ND
Otu000016	*Proteobacteria*(100)	*Alphaproteobacteria*(100)	*Rhodobacterales*(100)	*Rhodobacteraceae*(100)	*Sulfitobacter*(75)	4.43	Mb_ND
Otu000017	*Proteobacteria*(100)	*Alphaproteobacteria*(100)	*Rhodobacterales*(100)	*Rhodobacteraceae*(100)	*Celeribacter*(97)	6.81	Mm_ND
Otu000018	*Proteobacteria*(100)	*Alphaproteobacteria*(100)	*SAR11_clade*(100)	*Surface_1*(100)	*Candidatus_Pelagibacter*(93)	1.05	Mb_DT
Otu000019	*Proteobacteria*(100)	*Gammaproteobacteria*(100)	*Alteromonadales*(100)	*Colwelliaceae*(100)	*Thalassotalea*(100)	4.59	Mb_DT
Otu000020	*Proteobacteria*(100)	*Gammaproteobacteria*(100)	*Cellvibrionales*(100)	*Cellvibrionaceae*(100)	*Simiduia*(100)	5.83	Bb_DT

OTU ID	Phylum	Class	Order	Family	Genus	Max %	Micro_ID
Otu000021	*Bacteroidetes*(100)	*Flavobacteriia*(100)	*Flavobacteriales*(100)	*Flavobacteriaceae*(100)	*NS3a_marine_group*(100)	3.19	Mm_ND
Otu000022	*Proteobacteria*(100)	*Gammaproteobacteria*(100)	*Alteromonadales*(100)	*Alteromonadaceae*(100)	*Alteromonas*(95)	2.92	Mb_ND
Otu000023	*Bacteroidetes*(100)	*Flavobacteriia*(100)	*Flavobacteriales*(100)	*Flavobacteriaceae*(100)	*Polaribacter*(93)	2.46	Bb_DT
Otu000025	*Proteobacteria*(100)	*Gammaproteobacteria*(100)	*Pseudomonadales*(100)	*Pseudomonadaceae*(100)	*Pseudomonas*(89)	2.22	Mb_DT
Otu000027	*Proteobacteria*(100)	*Gammaproteobacteria*(100)	*Cellvibrionales*(100)	*Cellvibrionaceae*(100)	*Simiduia*(100)	2.70	Mb_ND
Otu000028	*Bacteroidetes*(100)	*Flavobacteriia*(100)	*Flavobacteriales*(100)	*Flavobacteriaceae*(100)	*Dokdonia*(94)	2.74	Bb_ND
Otu000029	*Proteobacteria*(100)	*Gammaproteobacteria*(100)	*Alteromonadales*(100)	*Pseudoalteromonadaceae*(100)	*Pseudoalteromonas*(100)	2.27	Mb_ND
Otu000031	*Proteobacteria*(100)	*Gammaproteobacteria*(100)	*Alteromonadales*(100)	*Colwelliaceae*(100)	unclassified(100)	1.52	Bb_ND
Otu000033	*Proteobacteria*(100)	*Deltaproteobacteria*(100)	*Desulfuromonadales*(100)	*GR-WP33-58*(100)	unclassified(100)	2.23	Bb_ND
Otu000034	*Bacteroidetes*(100)	*Flavobacteriia*(100)	*Flavobacteriales*(100)	*Flavobacteriaceae*(100)	unclassified(86)	1.17	Mm_ND
Otu000035	*Proteobacteria*(100)	*Deltaproteobacteria*(100)	*Desulfuromonadales*(100)	*GR-WP33-58*(100)	unclassified(100)	1.53	Mb_DT
Otu000038	*Bacteroidetes*(100)	*Flavobacteriia*(100)	*Flavobacteriales*(100)	*Flavobacteriaceae*(100)	*Leeuwenhoekiella*(100)	2.18	Mm_ND
Otu000039	*Proteobacteria*(100)	*Alphaproteobacteria*(100)	*Rickettsiales*(100)	*T9d*(100)	unclassified(100)	1.38	Mb_ND
Otu000041	*Proteobacteria*(100)	*Deltaproteobacteria*(100)	*Desulfuromonadales*(100)	*GR-WP33-58*(99)	unclassified(99)	2.05	Bm_DT
Otu000042	*Proteobacteria*(100)	*Gammaproteobacteria*(100)	*Alteromonadales*(100)	*Colwelliaceae*(100)	*Thalassotalea*(100)	1.19	Mb_ND
Otu000043	*Proteobacteria*(100)	*Alphaproteobacteria*(100)	*Rhodobacterales*(100)	*Rhodobacteraceae*(100)	*Citreicella*(80)	1.45	Mm_ND
Otu000088	*Bacteroidetes*(100)	*Cytophagia*(100)	*Cytophagales*(100)	*Flammeovirgaceae*(100)	*Reichenbachiella*(95)	1.75	Mb_DT
